# Toward an age of CRISPR delivery with non-viral biologics

**DOI:** 10.1016/j.omtn.2025.102822

**Published:** 2026-01-14

**Authors:** Joshua Tompkins, Roslyn M. Ray, Tristan A. Scott

**Affiliations:** 1 Department of Diabetes Complications, City of Hope – Arthur Riggs Diabetes and Metabolism Research Institute of City of Hope, 1500 E Duarte Rd, Duarte, CA 91010, USA; 2 CSL Behring, Novel Modalities, 225 Wyman St, Waltham, MA 02451, USA; 3 Center for Gene Therapy, City of Hope – Beckman Research Institute and Hematological Malignancy and Stem Cell Transplantation Institute at the City of Hope, 1500 E Duarte Rd, Duarte, CA 91010, USA

## Main text

A major goal in CRISPR/Cas gene therapy delivery is achieving biocompatible, transient delivery of gene editing machinery directly in patients. Among emerging delivery options, extracellular vesicles (EVs) hold promise as a new class of non-viral biologically derived vehicles for biotherapeutic applications for *in vivo* applications.^1^ Synthetic nanoparticles, such as lipid nanoparticles (LNPs), have empowered the non-viral delivery of CRISPR/Cas systems including a recent unprecedented advance in gene editing with the correction of a patient-specific genetic mutation in a rare disease (the “*N* = 1” patient).^2^ However, LNPs have been challenging to redirect to non-hepatic tissues and trigger unwanted adaptive immune responses as well as lipid-associated dose-limiting toxicity. Therefore, natural EVs could offer an alternative biocompatible delivery system for CRISPR/Cas delivery.

In this context, Pan et al. (2025) recently reported in *Science Translational Medicine* on the delivery of CRISPR/Cas protein complexes for therapeutic inactivation of pathogenic *Myo7a* variants associated with autosomal progressive hearing loss.^3^ Produced by virtually all cell types, EVs are native membrane-bound particles that transport DNA, RNA, and proteins to recipient cells. Small EVs (sEVs) or exosomes (between 50 and 200 nm in size) represent a promising non-viral platform for selective cargo loading and next-generation gene therapy delivery.^4^ Furthermore, their biocompatibility has been demonstrated in toxicity studies with high-level repeat dosing *in vivo* with no observed side effects.^5^ EVs, like LNPs, could protect naked Cas9 protein from degradation by serum enzymes and prevent immunogenicity toward the bacterially derived protein, allowing for possible repeat administrations. However, EVs face several challenges, especially efficient Cas9 protein loading into the luminal compartment of the EVs, efficient delivery into the cytosol of the recipient cell, and a scalable means of production for translation.

Pan et al. combined mesenchymal stem cell (MSC)-derived sEVs with an innovative high-throughput microfluidic droplet-based electroporation (μDES) to load EVs with Cas9 ribonucleoprotein (RNP) complexes. Cas9 RNPs were used, as they have reduced genomic off-target effects compared to other expression formats, such as DNA or mRNA. While MSC-derived EVs have anti-inflammatory and potentially tissue regenerative properties and have been previously applied clinically to attenuate inflammation in cochlear implantations.^6^ The μDES system achieved up to 80% Cas9 loading efficiency, a significant improvement over electroporation and lipofection, all while preserving EV physical characteristics (size and charge), the integrity of EV markers, and Cas9 cleavage activity.^7^^,^^8^

For *in vivo* testing, Pan et al. used a transgenic mouse model of *Myo7a*-associated hearing loss. *Myo7a* encodes a myosin with an essential role in the development of sensory hair cells and signal transduction; the heterozygous pathogenic shaker1 (sh1) murine model results in gradual hearing loss by 6 months. MSC Cas9 EVs resulted in successful gene editing and Cas9 localization within the organ of Corti.^3^ Of note, reporter-labeled Cas9 was detected in outer and inner hair cells when delivered by MSC EVs, whereas LNP-delivered Cas9 was distributed to nerve bundles, highlighting a unique biodistribution profile of EV delivery. Remarkably, gene editing improved hearing thresholds by approximately 20-decibel across the 4–32 kHz range as tested 6 months after treatment compared to control MSC EVs or LNP-delivered Cas9. Furthermore, the EV-Cas9 RNP treatment significantly reduced oxidative stress markers associated with genetic hearing loss. The effects are likely linked to the EV's unique distribution profile to this site and a possible advantage where synthetic nanoparticles fail. Overall, this study showed a critical proof of concept of a unique scalable loading solution of EVs to deliver CRISPR technology with disease-modifying capacity.

However, challenges remain. Related to efficiency *in vivo* gene editing, although there are notable successes in the liver and in bone marrow hematopoietic stem cells, generally *in vivo* gene editing is inefficient,^9^ requiring both proper cell targeting and specific Cas localization within the genome. In this study, the *in vitro* percentage of CRISPR/Cas-induced insertion-and-deletions (indels) was 5.24% using mDES-produced RNP MSC-EVs when delivering RNPs to primary isolated fibroblasts.^3^ Despite easy accessibility of the inner ear and good target tissue EV biodistribution, the percentage of indels in the organ of Corti was reported at ∼0.02% with MSC-EV RNPs. As noted, this editing was higher than LNPs or an alternative EV cell source used in the study derived from a mouse auditory cell line. It is unclear how this low gene editing efficiency resulted in a greater than 2-fold drop in Myo7a^Sh1^ expression and significant functional hearing recovery in mutant mice. However, the authors also reported impressive reductions in inflammatory markers likely mediated from MSC-EV activities.^3^ Other examples illustrate how relatively inefficient gene editing can provide significant functional benefits. For example, in several blood disorders, hematopoietic stem and progenitor cell (HSPC) gene editing, even at very low efficiency, can provide significant functional benefits over time from clonal outgrowth of gene-edited stem cells.^10^ Additional mechanistic insights into how MSC EV and CRISPR-mediated gene editing in Myo7a-associated hearing loss will catalyze translation of such findings into human trials.

Though no trials currently utilize combinations of EVs and CRISPR/Cas or EVs as a delivery vehicle for gene editing machinery, examples of efficient EV loading like these published by Pan et al. help pave the way for these future iterations. Looking forward, improving *in vivo* genome editing efficiencies with the EV-delivered CRISPR should broaden the number of genetic conditions that may be suitable for clinical correction. Considerable work also remains for viable drug product development, including improving long-term EV circulation *in vivo*, delivery to low accessibility tissue, large-scale manufacturing and purification of EVs, and the stability of engineered EV products. Nevertheless, many EVs, especially stem-cell-derived EVs, have natural anti-inflammatory, immune-privileged, and tissue-restorative roles, which can be engineered to specifically enhance the tropism of their beneficial cargos. Therefore, EVs may offer a protective advantage to cells specifically undergoing EV-Cas9-associated DNA damage responses. As a unique feature of sEV biology, these particles can access biologically challenging regions when administered systemically, such as the blood-brain barrier (BBB) crossing and may offer new opportunities in currently inaccessible tissues for treatment. We ultimately expect future clinical trials to include EVs for the delivery of gene editing machinery across several genetic conditions.

Various EV biologics are on the horizon with emerging industry investment focusing on exosomes as well as engineered virus-like particles and protein delivery vehicles for efficient delivery of gene editing cargo into target cells (Table 1). This investment is driven by the need for alternative, less immunogenic, and efficient *in vivo* extra-hepatic delivery vehicles for tissue targets that show limited success with LNP-based delivery strategies or require the need for short transient exposure to gene editing cargo. Of note is EvoX Therapeutics with several pre-clinical projects delivering RNPs via exosomes for CNS indications (Table 1), highlighting the future is bright for engineered EVs in biomedical applications.

**Table 1:** Industry investment into gene-editing cargo delivered using engineered EVs

Name	Platform type	Technology focus	Key applications	Clinical stage	Total funding (USD)
Aera Therapeutics	Protein nanoparticles	Protein-based nanoparticle delivery	Gene editing and RNA therapeutics for CNS	Discovery	Combined Series A + Series B total $193M (Feb 2023)
Capricor Therapeutics	Exosomes	StealthX Therapeutic exosomes, bioengineered with surface receptors and/or loaded with siRNAs	Vaccines and other	Discovery (other); phase 1 for vaccines (NCT07095231)	Notable 2024 capital raise ∼$69M (private placement + ATM); multiple other post-IPO raises
Evox Therapeutics	Exosomes	DeliverEX and ExoEdit platforms (delivery of gene editing cargo via exosomes)	CNS diseases: Huntington disease, SCA2 and ALS	Preclinical	Total funding ∼$156M; Series C $95M (Feb 2021)
Azalea Therapeutics	Virus-like particles (EDVs)	Antibody-targeted enveloped delivery vehicles (EDVs) delivering Cas9 RNPs to T cells; dual-vector with AAV HDR template	*In vivo* CAR-T (B cell malignancies; autoimmune); solid tumors	Preclinical (NHP data); first-in-human planned 12–18 months	$82M total (Seed + Series A; Series A $65M closed Nov 2025)
GeneEditBio	Protein nanoparticles	Protein delivery vehicle CRISPR-Cas RNP editing for TGFBI corneal dystrophy	TGFBI corneal dystrophy (GEB-101); intrastromal injection	FIH open-label and dose escalation clinical study	PitchBook shows $34M raised; includes $25M (Apr 2022) and $9M (Nov 2021); later rounds undisclosed
nChroma Bio	Non-viral particles + epigenetic editors	Programmable epigenetic editing cargo with Nvelop’s non-viral delivery for *in vivo* therapies	Hematology (HSPC, T/B cell targeting)	Pre-clinical and discovery	$75M financing on formation (Dec 2024) via merger of Chroma Medicine and Nvelop Therapeutics

Co-pilot assisted in the generation of the table and provided the funding data using the sources. Amounts reflect publicly reported venture rounds (or post-IPO raises where relevant) as of Dec 1, 2025. Sources are included per row and were accessed via company press releases, investor pages, and third-party databases (Tracxn, PitchBook, Business Wire, and FirstWord).

## Declaration of interests

The authors declare no competing interests.
